# Antimicrobial Resistance in Streptococcus pneumoniae, Taiwan

**DOI:** 10.3201/eid0812.020178

**Published:** 2002-12

**Authors:** Po-Ren Hsueh, Kwen-Tay Luh

**Affiliations:** * National Taiwan University Hospital, National Taiwan University College of Medicine, Taipei, Taiwan

**Keywords:** antimicrobial resistance, Streptococcus pneumoniae, Taiwan

## Abstract

Taiwan has one of the highest levels of antibiotic-resistant pneumococcus in the world. Pneumococcal isolates not susceptible to penicillin first appeared in Taiwan in 1986; in 1995 an increase in the prevalence of nonsusceptibility to penicillins, extended-spectrum cephalosporins, trimethoprim-sulfamethoxazole, and macrolides as well as multidrug resistance began to be recognized. With the persistence of antibiotic selective pressure, resistance in some antibiotics reached a high plateau (β-lactam antibiotics) or continued to increase (macrolides), while novel resistance (fluoroquinolones) emerged in the last 3 years. Widespread distribution of some novel resistant 23F and 19F clones (and the international epidemic of 23F clones) contributes further to the rapid increase of resistance. Because Streptococcus pneumoniae is a major pathogen that causes community-acquired lower respiratory tract infections and meningitis in adults and children, antibiotic-resistance in this organism is a serious problem.

For more than a century, Streptococcus pneumoniae has been known as the major bacterial pathogen in humans, causing substantial illness and death (1,2). Before 1967, this organism was uniformly susceptible to penicillin. In the early 1990s, pneumococcal isolates appeared that exhibited a high level of resistance to penicillin and other β-lactam antibiotics (1). The widespread emergence of this resistance in many countries has become a major concern in recent years. The persistence of high antibiotic selective pressure in the community and international spread of epidemic or countrywide circulation of endemic multiresistant clones have substantially contributed to the crisis of resistance (3). This resistance has complicated treatment options and increased the likelihood of treatment failure (2).

The Asian region is one of the epicenters for pneumococcal resistance, and Taiwan has become the focus of pneumococcal resistance since 1996, particularly after several reports documented the alarmingly high prevalence among clinical isolates of resistance to β-lactam antibiotics and macrolides (4–12). The Center for Disease Control under the Department of Health in Taiwan established an active surveillance program in 1998 to study the epidemiologic features of invasive pneumococcal diseases in Taiwan. Furthermore, a nationwide surveillance system for antimicrobial resistance involving 12 major teaching hospitals (Surveillance from Multicenter Antimicrobial Resistance in Taiwan [SMART]) has also tracked the trends of pneumococcal resistance annually since 2000. In this report, we will discuss the trends of pneumococcal resistance, evidence of dissemination of resistant clones, and substantial community use of antibiotics, and highlight critical resistance problems.

## Disease Burden and Severity

 The incidence of invasive infections caused by S. pneumoniae in Taiwan is still unknown, although several studies regarding invasive pneumococcal infections in adults and children have been reported (9,13–19). The overall mortality rate (42.5%) for elderly patients (>65 years of age) with invasive infections (bacteremia, pneumonia, pleural empyema, meningitis, septic arthritis, and peritonitis) was higher than the rates for patients 19–64 years of age (22.4%) and for children (8.1%) (9). Some factors, such as the presence of serotype 3 strain, shock as initial presentation, and multilobar pneumonia, were significantly associated with death from invasive infections (14,15). Another report from central Taiwan indicated that the overall mortality rate for children with invasive pneumococcal infections was 20.3%, and in 53.3%, the infections progressed rapidly to death (18). Other studies found that 70% to 80% of adult patients with invasive pneumococcal disease had underlying diseases (malignancies, followed by congestive heart failure and diabetes mellitus) (14,15). Patients with HIV infection or multiple myeloma in whom invasive pneumococcal infection developed were extremely rare (14,13–20). No significant difference was found in the mortality rates of patients with penicillin-susceptible (PSSP) and those with penicillin-nonsusceptible S. pneumoniae (PNSSP) infections (9,14,15,18).

The prevalence of this organism that causes community-acquired pneumonia or meningitis in adults or children is obscure. Several reports have indicated that this organism causes 19% to 33% of infections that results in bacterial meningitis in children (21–23). In one study of adult patients, 28% of the community-acquired bacterial meningitis were caused by S. pneumoniae, the highest proportion after that was by Klebsiella pneumoniae (33%) (24). The incidence (cases per 100,000 emergency visits) of pneumococcal meningitis at National Taiwan University Hospital was 2.8 in 1997–1998 (24). In other study, S. pneumoniae accounted for 21.8% of bacterial pathogens isolated from middle ear fluid from 243 children with acute otitis media (25).

## Antimicrobial Resistance

### Resistance to Penicillin and Other β-lactam Antibiotics

**Figure 1:**
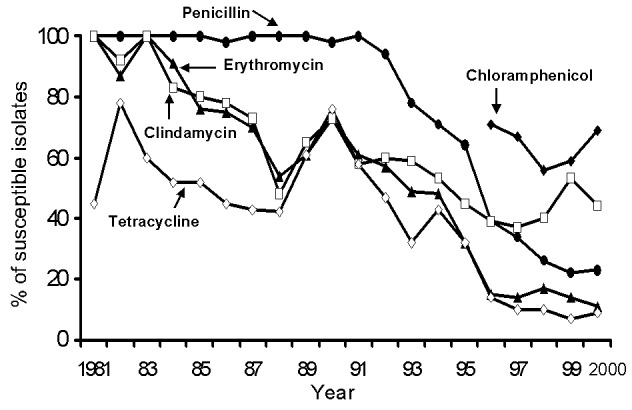
Prevalence of susceptibility to five antimicrobial agents for *Streptococcus pneumoniae* isolates at the National Taiwan University Hospital, 1981–2001. Susceptibility testing was performed with the disk diffusion method. For penicillin susceptibility testing, the 10-U penicillin disk was used from 1981 to 1989, and the 1-μg oxacillin disk since 1990.

The first clinical isolate of S. pneumoniae not susceptible to penicillin (MIC, >2 μg/mL) was reported in 1986 (26). At National Taiwan University Hospital, a clinical isolate of PNSSP might have also been first seen in 1986 (the disk used for determining penicillin susceptibility was not the standard 1-μg disk). A stepwise decline in the annual rates of susceptibilities to penicillin from 1981 to 2000 at National Taiwan University Hospital (disk susceptibility data from all sites of isolates) (Figure 1) and a high prevalence (60%–84%) of clinical PNSSP isolates were noted throughout the island (disk susceptibility data from all sites of isolates) (12).

The table summarizes the results of dilution susceptibility tests for S. pneumoniae isolated from various clinical specimens (6–8, 10) and normally sterile sites (9) of patients seen from 1996 to 2000. The results were similar to those determined by the disk diffusion method (12). The proportions of high-level resistance to penicillin (MIC >2 μg/mL) varied from 8% to 33%; however, isolates exhibiting an MIC of penicillin of <4 μg/mL accounted for <12%. About 60% of isolates with intermediate resistance to penicillin also had intermediate resistance to cefotaxime or ceftriaxone, and nearly all isolates resistant to penicillin were also not susceptible to those two agents (6–9). The resistance level was higher among nasopharyngeal isolates from colonized children (71%) or among isolates from children with invasive conditions (such as bacteremia, pneumonia, meningitis, peritonitis, and empyema thoracis) than those from adults with invasive conditions (76% vs. 45%) (chi-square test, p=0.0000009) (5,9). About 50% of isolates from blood or cerebrospinal fluid samples were not susceptible to penicillin ( 6,7,9). Among isolates recovered from patients hospitalized in the intensive care units from five major teaching hospitals in Taiwan, 58% were not susceptible to penicillin, 33% were not susceptible to cefotaxime, and 21% showed intermediate resistance to imipenem (10).

**Table:** Summary of dilution susceptibilities for *Streptococcus pneumoniae* isolates, Taiwan, 1996–1999

	% of nonsusceptible isolates (intermediate/resistant)				
Antimicrobial agent	1996–1997^a^	1996–1997^b^	1998–1999^c^	1998–1999^d^	2000^e^
	(n=200)	(n=550)	(n=267)	(n=288)	(n=24)
Penicillin	61 (28/33)	56 (43/13)	76 (51/25)	56 (34/22)	58 (50/8)
Amoxicillin (-clavulanate)		50 (34/16)	33 (32/1)	22 (12/10)	—
Cefuroxime	—	—	67 (16/51)	43 (6/37)	—
Ceftriaxone (cefotaxime)	39 (16/23)	13 (11/2)	56 (54/2)	25 (15/10)	33 (29/4)
Cefepime	43 (19/24)	—	—	—	42 (21/21)
Imipenem	—	15 (13/2)	—	14 (14/0)	21 (21/0)
Meropenem	—	—	—	—	4 (4/0)
Erythromycin	83 (6/77)	74 (5/69)	—	76 (4/74)	—
Azithromycin	—	78 (4/74)	94 (4/90)	78 (6/72)	—
Clarithromycin	90 (9/81)	—	95 (6/89)	—	—
Trimethoprim- sulfamethoxazole	87 (6/81)	—	65 (33/32)	71 (30/41)	—
Ciprofloxacin^f^	2	—	4	—	0
Levofloxacin		—	1 (0/1)	—	0
Moxifloxacin	0	—	1 (0/1)	—	0
Rifampin	0	7 (7/0)	—	—	—
Vancomycin	0	0	—	0	0
Quinupristin-dalfopristin	—	—	8 (6/2)	—	42 (38/4)
Linezolid	—	—	0	—	0

^a^Data adopted from reference 6. All isolates were recovered from various clinical specimens (60% of isolates from respiratory secretions and 27% from blood samples) of patients treated at the National Taiwan University Hospital (NTUH). ^b^ Data adopted from references 7. All isolates were recovered from various clinical specimens (36.7% from sputa and 42.3% from sterile sites) of patients treated at 14 major hospitals in Taiwan. ^c^Data adopted from references 8. All isolates were recovered from various clinical specimens, including 86.4% from respiratory secretions and 8.8% from sterile sites (cerebrospinal fluid, blood, ascites and pleural fluid) of patients treated at five teaching hospitals in Taiwan. ^d^Data adopted from reference 9. All isolates were recovered from normally sterile sites (cerebrospinal fluid, blood, ascites and pleural fluid) of patients treated at all major hospitals in Taiwan. ^e^Data adopted from reference 10. All isolates were recovered from various clinical specimens (12 isolates were recovered from non-respiratory secretions) of patients treated at five teaching hospitals in Taiwan. ^f^Ciprofloxacin MIC >4 μg/mL.

### Resistance to Macrolides

A similar trend of decreased annual rates of susceptibility for S. pneumoniae was also observed in erythromycin. (Table and Figure 1). Overall, the prevalence of clinical isolates of S. pneumoniae not susceptible to erythromycin was 67% to 100% island-wide (12). Only about one-third of the erythromycin-resistant isolates exhibited M-phenotype (27).

### Resistance to Fluorquinolones or Other Antibiotics

Isolates not susceptible to ciprofloxacin (MIC >4 μg/mL) might have first been noted in 1996, but the first clinical isolate that was highly resistant to ciprofloxacin (MIC >32 μg/mL) and other newer fluoroquinolones was documented in 1999 (6,8,28,29). This isolate had mutations in genes gyrA and parC and also possessed an efflux mechanism (28). Isolates not susceptible to trimethoprim-sulfamethoxazole accounted for 60% to 90% (6–10). All isolates are susceptible to vancomycin and linezolid, but some strains were resistant to quinupristin-dalfopristin (10,29). More than 90% of PNSSP isolates were also resistant to multiple antibiotics (resistant to at least three classes of antibiotics) (6–10).

Several factors limit our conclusions regarding the trends of pneumococcal resistance in Taiwan. First, data on antibiotic resistance gained from disk diffusion testing in some studies are not optimal because such studies tend to overestimate penicillin resistance compared with estimates determined by the dilution method. Second, studies that report resistance data of a mixture of invasive and noninvasive isolates and of isolates from different location for different years make it hard to assess the trends over time since resistance may vary with sites of isolates and geographic location. Third, resistance data from one hospital cannot be compared with data from larger populations since single hospitals may have more referral bias for severe cases and do not necessarily represent the phenomenon of the larger population.

### Spread of Resistant Clones

 Although one report has supported the idea that the international epidemic (Spanish 23F) clone was introduced and diffused in Taiwan (30), multiple domestic and novel clones (23F, 19F, and 6B), which have exhibited high-level resistance to penicillin, extended-spectrum cephalosporins, and macrolides, continuously circulate in our community (9,31,32). Two penicillin-resistant clones that acquired separate mechanisms of macrolide resistance, i.e. Taiwan-23F clone isolates exhibiting high-level resistance to erythromycin with an MIC of >256 μg/mL (ermAM genes mediated) and Taiwan-19F clone isolates with erythromycin resistance shown by an MIC of 1.5–8 μg/mL (mefE gene–mediated), have already spread (30). Isolates belonging to the same clone caused various invasive diseases of different patients who lived in different parts of Taiwan, and resulted in bacteremic pneumonia in siblings (9,31,33). The spread of an endemic and highly resistant 23F clone (penicillin MIC, 4 μg/mL and erythromycin MIC, >256 μg/mL) in one day care center was also reported (34). The spread of Taiwan clones to other parts of the world (Taiwan 19F-14 and Taiwan-6B clones to Hong Kong and United Kingdom; Taiwan 19F-14 to the United States) has also been documented (30, 35, 36). Although the dissemination of fluoroquinolone-resistant strains has been documented in Hong Kong and Brooklyn, New York (37,38), to date, the clonal spread of these resistant strains has not been found in Taiwan.

## Impact of Resistance on Antimicrobial Therapy

 A high dose of intravenous penicillin G is still recommended as one of the primary drugs of choice for the empirical treatment of patients with community-acquired pneumonia who need hospitalization because about 10% of our clinical isolates have an MIC of penicillin of ≥4 μg/mL (6–10,39). MIC testing, mostly by the E-test, for penicillin-nonusceptible (oxacillin 1-μg disk) isolates recovered from respiratory secretion or blood specimens from patients without meningitis, is always performed in most teaching hospitals in Taiwan (39, 40). Obviously, the guidelines for the treatment of community-acquired pneumonia in adults recommended by the American Thoracic Society in 2001 (41) and the Infectious Diseases Society of America in 2000 (42), as well as suggestions for the management of sinusitis, otitis media, and chronic bronchitis, which include one macrolide alone as the drug of choice or as an alternative antimicrobial agent, are inappropriate in Taiwan. This is because of the high incidence of macrolide resistance and the high proportion of MLSB-phenotype among these resistant isolates.

## Serotype Prevalence and Vaccine Coverage

**Figure 2:**
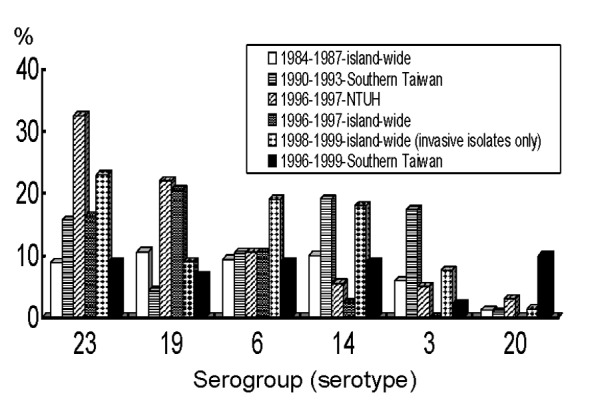
Distribution of six major serogroups or serotypes of clinical isolates of *Streptococcus pneumoniae*, Taiwan, 1984–1998.

Figure 2 illustrates the distribution of six major serogroups or serotypes of S. pneumoniae isolates from 1984 to 1998 (4,6,7,9,19,43). The frequencies of serogroups (serotypes) 23 (23F) and 19 (19F) increased remarkably, whereas those of serotypes 14, 3, and 1 declined. Some unusual serotypes (serotypes 20, 11, 7, and 8) appeared to emerge in southern Taiwan. No relationship was found between the serogroup or serotype distribution of isolates and the patients’ ages (p >0.05) (6,7,9). PNSSP exhibited resistance to isolates from various serogroupsor serotypes. Among them, serogroups 11 and 23 isolates had the highest incidence of penicillin nonsusceptibility and multidrug resistance. However, serotype 3 isolates (0%–22%) and serotype 20 (11%) isolates had lower rates of nonsusceptibility to penicillin (4,6,9,19). In general, more than 90% of isolates causing invasive infections were included in the serogroups or serotypes covered by the 23-valent pneumococcal and 7-valent conjugate vaccine (4,9).

## Extent of Antibiotic Use in the Community

Before 1995, few regulations regarding antibiotic use existed for physicians in primary care clinics or hospitals. Furthermore, many antibiotics could easily be obtained at drugstores without a prescription. Although the medical payment has been regulated by the National Health Insurance program, implemented in Taiwan in 1995, antibiotics are still commonly used and seem to be overprescribed in primary care units (44). From 1996 to 1999, about 12% to 14% of total patient-visits in primary care units had antibiotic use. The common cold (32.3%) was the most frequent diagnosis for which antibiotics were prescribed. Penicillins (35.4%), cephalosporins (26.5%), and macrolides (21.6%) were the most commonly prescribed classes of antibiotics (44). In 1999, a reported point prevalence rate of about 7.5% among healthy high school students and healthy ambulatory elderly persons who had antimicrobial activity in their urine suggested that this may be the baseline for antibiotic use in the community (45). Surprisingly, over half of the patients who came to the emergency department of a large teaching hospital in Taiwan had taken antibiotics within the previous 12–48 hours. With the increasing and highly selective pressure of antibiotic usage in our community, the crisis of resistance continues to exist.

In the new millennium, the Center for Disease Control under the Department of Health in Taiwan has made nationwide surveillance of antimicrobial resistance and strict control of antibiotic usage major tasks. The new regulations for antibiotic prescription, established by the Bureau of National Health Insurance in 2001 (44), restrict the inappropriate use of the so-called first-line antibiotics (first-generation cephalosporins, macrolides, and gentamicin) for treating various infections, particularly the trivial upper respiratory tract infection. The use of the 23-valent pneumococcal vaccine, especially among high-risk adults and older persons, is encouraged, and new protein-conjugate vaccines will be introduced in the near future.

## Conclusion

 Taiwan has one of the highest levels of antibiotic resistant pneumococcus in the world. With the increase in international travel, the interchange of resistant clones among countries is unavoidable and the widespread distribution of these clones is expected. Strategies to limit the upsurge of resistant pneumococcus include improved surveillance, reduced antibiotic usage, and increased vaccination of persons at high risk. The judicious use of antimicrobial agents is necessary to avoid providing a selective advantage for resistant organisms. The active surveillance of resistant organisms can help track local resistance problems, prevent the future dissemination of these organisms, and provide options appropriate for empiric therapy.
